# Calcitermin-Loaded Smart Gels Activity against *Candida albicans*: A Preliminary In Vitro Study

**DOI:** 10.3390/gels9020165

**Published:** 2023-02-18

**Authors:** Denise Bellotti, Maria D’Accolti, Walter Pula, Nicolas Huang, Fanny Simeliere, Elisabetta Caselli, Elisabetta Esposito, Maurizio Remelli

**Affiliations:** 1 Department of Chemical, Pharmaceutical and Agricultural Sciences, University of Ferrara, I-44121 Ferrara, Italy; 2 Faculty of Chemistry, University of Wrocław, F. Joliot-Curie 14, 50-383 Wrocław, Poland; 3 Institut Galien Paris-Saclay (CNRS UMR 8612), Faculté de Pharmacie, Bâtiment Henri Moissan, Université Paris-Saclay, 91400 Orsay, France

**Keywords:** calcitermin, *Candida albicans*, hydrophilic gel, xanthan gum, poloxamer, controlled release

## Abstract

Calcitermin is an antimicrobial peptide of 15 amino acids found in human nasal fluid characterized by antifungal and antibacterial properties. *Candida albicans* is the most common human fungal pathogen affecting many tissues, such as vaginal mucosa. In this study a formulation suitable for calcitermin administration on vaginal mucosa was developed for the treatment of fungal infections. To favor topical application, mucosal adhesion, and permanence, gels based on poloxamer 407 and xanthan gum were designed and compared with regard to their rheological behavior, erosion, and leakage. The selected gel was loaded with calcitermin, whose release kinetic was evaluated in vitro by Franz cells. An antifungal activity assay was conducted to assess the calcitermin anticandidal potential and the effect of its inclusion in the selected gel. The rheological study revealed the elastic and viscous moduli behavior as a function of poloxamer 407 and xanthan gum concentration. Xanthan gum presence decreased the transition temperature of the gel, while prolonging its erosion and leakage. Particularly, poloxamer 407, 18% and xanthan gum 0.4% were chosen. The calcitermin loading in the selected gel resulted in a transparent and homogeneous formulation and in a 4-fold decrease of the release rate with respect to the calcitermin solution, as evidenced by Franz cell study. The anticandidal activity tests demonstrated that calcitermin-loaded gel was more active against *Candida albicans* with respect to the peptide solution.

## 1. Introduction

Currently, the increasing need of innovative pharmacological therapies against infectious diseases is unquestionable. Despite the improvement of antimicrobial agents, the phenomenon of antimicrobial resistance—the resistance developed by pathogenic microorganisms against previously effective treatments—represents an ever-present clinical and financial burden for the global healthcare system [1,2]. Among several novel approaches devoted to overcoming the global antimicrobial resistance crisis, the use of antimicrobial peptides represents a promising strategy for the design of new drugs [3,4,5,6]. Antimicrobial peptides are phylogenetically ancient biomolecules with a broad spectrum of activity. They are effective against a wide variety of pathogens and are present in all living organisms (invertebrates, vertebrates, plants, and prokaryotes) [6]. Moreover, several studies have shown that divalent metal ions, such as Zn^2+^, can modulate the efficacy of antimicrobial peptides [7,8,9,10], for example, by modifying their physicochemical properties (e.g., charge or structure) [11,12].

**Figure 1:**
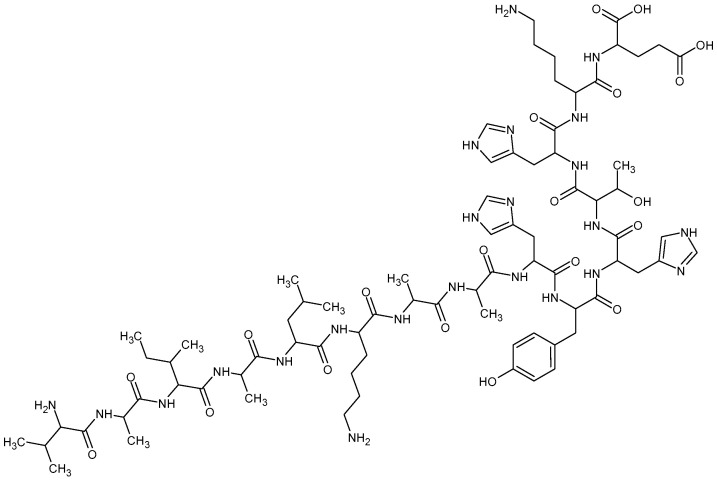
Primary structure of Cal (VAIALKAAHYHTHKE).

Calcitermin (Cal) is an antimicrobial peptide consisting of 15 amino acids (VAIALKAAHYHTHKE, Figure 1), found in human nasal fluid. It corresponds to the C-terminal domain of calgranulin C, a pro-inflammatory protein of the S100 family [13].

The acid–base properties of Cal have been extensively studied, it is soluble in water solutions and in a wide pH range (2–11) [14]. Moreover, the thermodynamics, structure and coordination chemistry of zinc– and copper–calcitermin complexes have been thoroughly investigated [14]: Cal is a very good chelator for both Zn^2+^ and Cu^2+^ ions and it mostly binds them through the three alternated histidine residues in position 9, 11, and 13. Circular dichroism measurement revealed that Cal and its metal complexes are able to assume an α-helical conformation in structure-promoting membrane mimetics, wheeas in aqueous solution they prevalently have a random coil structure [13,14]. Some preliminary studies revealed that Cal exhibits antifungal and antibacterial properties under acidic conditions, especially in the presence of Zn^2+^ and Cu^2+^ ions [14,15]. Such bioactivity prompted us to deepen the properties of Cal as potential antifungal agents against *Candida albicans (C. albicans)*. This pathogen is considered the fourth most frequent cause of fungal infections, especially affecting vaginal and oral mucosae [16,17]. In immunocompromised patients, this opportunistic yeast species can be responsible for life-threatening infections, causing extensive mucosal colonization and local and/or systemic disease, until it leads to severe candidiasis and bloodstream infections (candidemia) [18,19]. Azoles, such as fluconazole, ketoconazole, voriconazole, itraconazole, and clotrimazole, are the first-line drugs to treat these mycoses, although they often induce antimicrobial resistance [20,21]. The topical antifungal drugs administration offers several advantages in the treatment of superficial mycoses, such as the prevention of the drug systemic toxicity, as well as the efficacy at the local level, due to the direct application at the site of infection [22,23].

Aiming at finding a new topical strategy in the treatment of *C. albicans* infections, we designed a smart hydrophilic gel constituted of biocompatible and biodegradable polymers, to obtain a vehicle suitable for vaginal administration of Cal. An ideal gel for mucosal administration should (i) be scarcely viscous at room temperature, enabling reproducible administration, and (ii) undergo gelation at body temperature, maintaining its permanence on the mucosa surface to sustain drug release [22,23,24]. In this respect, in the present investigation, a gelified system was specifically designed, based on poloxamer 407 (p-407) and xanthan gum (x-gum). P-407 is a biocompatible non-ionic poly(oxyethylene)poly(oxypropylene) (PEO-PPO) block copolymer that can self-assemble in water-forming micelles, behaving like a surfactant, and displays a thermo-reversible character upon dispersion in water. Indeed, as a function of its concentration, p-407 undergoes a sol–gel transition phenomenon, above a T_sol-gel_ transition temperature, increasing its viscosity due to a close micelle packing [25,26]. This smart behavior makes p-407 micellar solutions suitable for mucosal administration, being easily administrable below their transition temperature, while turning into semisolid forms when approaching body temperature [27,28]. In situ gel forming systems are particularly suitable for vaginal administration, being characterized by easy application, due the low viscosity of the formulation, and by the subsequent gelation phenomenon [22]. The biodegradable character of these gels avoids the need of the formulation removal, while the gel viscosity allows a long permanence of the loaded drug on the vaginal mucosa [23]. In this respect, to treat *C. albicans* infections, some authors previously proposed p-407-based gels to administer clotrimazole, fluconazole, econazole, oxiconazole, or amphotericin B on skin and vaginal mucosa [29,30,31,32,33,34,35,36].

Notwithstanding the advantages of p-407 based forms, their rapid dissolution in aqueous media, such as the vaginal fluid, results in short residence time [37,38]. At this regard, other mucoadhesive polymers, such as hydroxypropyl methylcellulose, and x-gum, can be added to p-407 to improve the formulation residence time [39,40,41,42,43,44]. X-gum is a natural heteropolysaccharide, produced by fermentation of the gram-negative bacterium *Xanthomonas campestris*. X-gum is widely employed for pharmaceutical applications, due to its biocompatibility and capability to produce transparent and stable gels upon dispersion in water [45]. In previous studies about the association of natural gums with p-407 in water, x-gum resulted in the most potent and efficient viscosity enhancer. Particularly, a drug delivery system based on p-407, x-gum, and guar gum was developed as in situ gel-forming eye drops [40]. In another investigation, p-407, 17% was used in association with x-gum 0.125–0.5%, confirming the capability of x-gum to increase the gel viscosity while retaining its thermosensitive characteristics [41].

In the present investigation, x-gum was added to p-407 to modulate its gelling behavior, while conferring bioadhesive properties to the gel. Rheology, erosion, and leakage tests enabled the influence of x-gum and p-407 on the gels to be evaluated. Cal was then loaded into the selected gel, and Franz cells associated with the nylon membrane were employed to evaluate Cal release kinetics. Remarkably, the anticandidal activity of Cal in solution or loaded in the gel was studied against the *C. albicans* ATCC 10231 strain.

## 2. Results

### 2.1. Gel Formulative Study

A formulative study was conducted to obtain a suitable semisolid formulation for mucosal administration. We chose to employ p-407 for its in situ gelling potential, while adding x-gum to ensure a long permanence on the vaginal mucosa in contact with body fluids. Previous studies showed that below 15% *w*/*w*, p-407 solutions undergo transition to gels at temperatures above 60 °C, whereas p-407 solutions above 18% *w*/*v* convert into stiff gels at 20–25 °C [28,29,30,31,32,33,34,35,36,37,38,39]. Thus, we solubilized p-407 15 and 18% *w*/*w* in lactate buffer (G15 and G18, respectively), to obtain pH 4.5 ± 0.1, compatible with vaginal milieu [46]. After complete solubilization of P-407, resulting in G15 or G18 free flowing solutions, x-gum 0.1–0.5% *w*/*w*, was added. All formulations appeared transparent and homogeneous after the x-gum addition, apart from x-gum 0.5%, *w*/*w*. Indeed, this x-gum concentration prevented its dispersion in G15 and G18, for this reason x-gum 0.4% *w*/*w* was selected as the upper limit (Table 1).

#### 2.1.1. Rheological Characterization

A preliminary visual inspection by the tube inversion method [47] enabled us to gain information about the x-gum influence on gel viscosity. As previously found, even the smallest addition of x-gum led to significant changes [41]. Namely, the higher the P-407 and x-gum concentrations, the lower the gel capability to flow, in particular G18/0.4 did not flow at all upon vial inversion (Figure S1). In this respect, it is expected that a viscous inelastic sample (sol) displays appreciable flow, whereas a sample having a yield stress (gel) does not flow [48].

**Figure 2:**
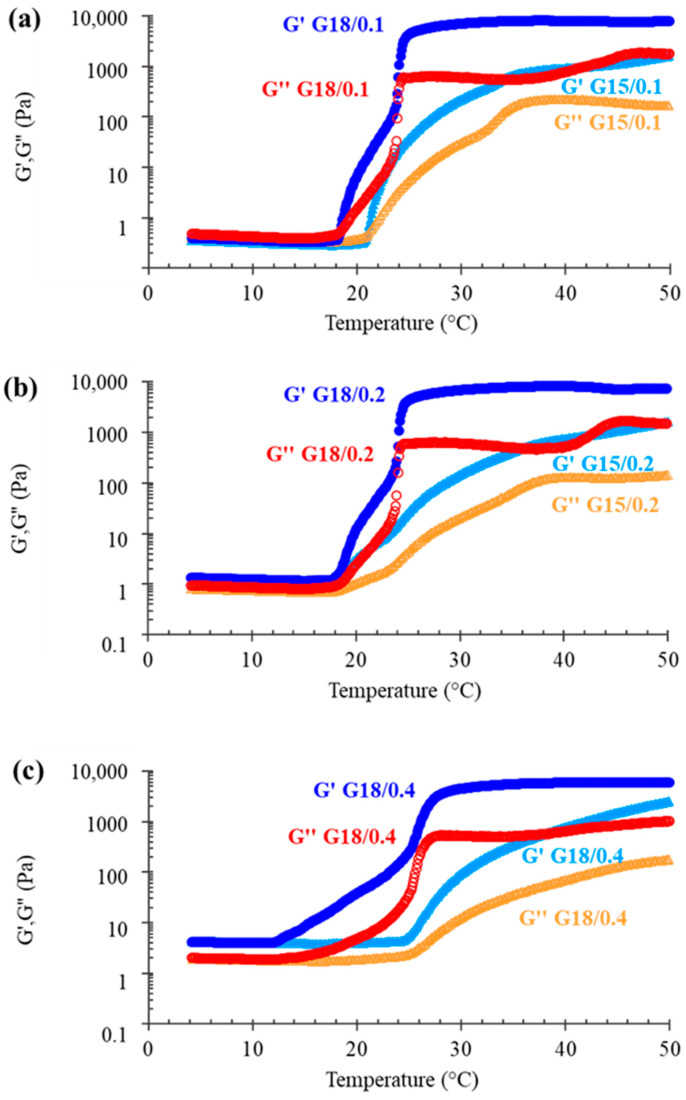
Variation of elastic G′ and viscous G″ moduli as a function of temperature; analyses were performed on gels based on p-407 15 or 18% and x-gum 0.1 (**a**), 0.2 (**b**), or 0.4 (**c**) %, *w*/*w*.

In order to better investigate the capability of x-gum to decrease p-407 gelation temperature, the gel rheological viscoelastic behavior was studied, measuring the elastic modulus G′ and the viscous modulus G″. The former indicates the stored elastic energy reflecting the solid-like component of elastic behavior, whereas the latter indicates the dissipated loss of energy, reflecting the loss or viscous modulus [28]. Figure 2 displays the the G15 and G18 variation of elastic and viscous moduli G′ and G″, as a function of temperature, under the addition of x-gum 0.1, 0.2, or 0.4%, *w*/*w*, and Table 2 reports the obtained rheological parameters.

The addition of x-gum led to an increase of the elastic G′ and viscous G″ moduli before the gelation temperature. The more x-gum, the higher the increase, especially of G′. In the case of G15/0.1 and G18/0.1, at a well-defined temperature (T_sol-gel_), a sudden increase of the moduli was observed, with G′ becoming higher than G″, indicating a transition from liquid (sol) to structured (gel) form. After gelification, G′ and G″ increased drastically, p-407 being mainly responsible for the viscoelasticity of the samples. In the case of 0.2% and 0.4% x-gum, the transition temperature T_sol-gel_ was determined at the onset of a sharp G′ increase, since the G′-G″ crossover was not appreciable, whereas G′ was always higher than G″. The lowest value of T_sol-gel_ (18.6 °C) was found in the case of G18/0.4, followed by G18/0.2 and G18/0.1 (Figure 2 and Table 2), suggesting that x-gum promoted the gelification process.

#### 2.1.2. Erosion Evaluation

**Figure 3:**
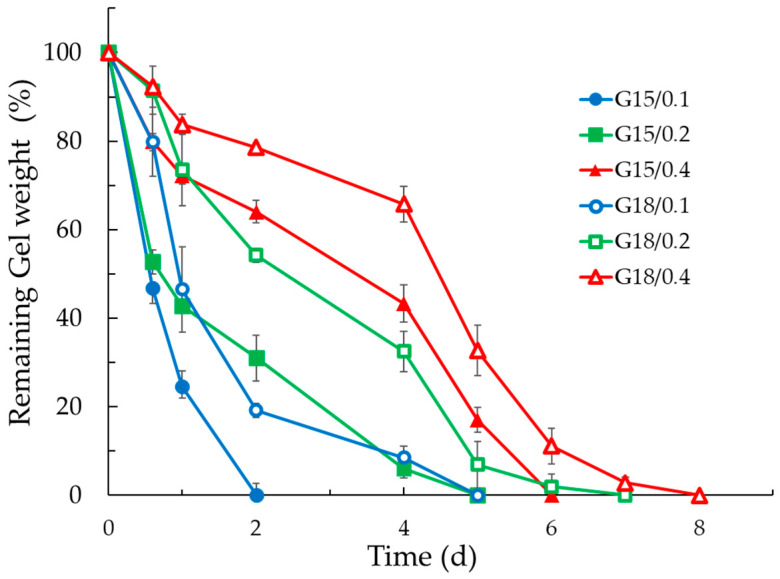
Erosion profiles of G15/0.1, G15/0.2, G15/0.4, G18/0.1, G18/0.2, and G18/0.4 at 37 °C. Results are shown as the percentage of remaining gel weight as a function of time. Data are the mean of three experiments ± s.d.

In order to obtain information about the residence time of the gels in the vaginal cavity, erosion tests were performed using a solution that simulates the vaginal fluid (SVF), pH 4.5, at 37 °C. Notably, the p-407 micellar packing arrangement can rapidly dissociate in the presence of excess aqueous media, thus leading to the gel matrix degradation [37]. Figure 3 reports the results, expressed as percentage of remaining gel weight, as a function of time.

The results showed that x-gum concentration governed the erosion profiles. Indeed, the faster erosion rates were observed in the case of the lower x-gum concentration, with complete erosion occurring in 2 d in the case of G15/0.1 and 5 d in the case of G18/0.1. Conversely, in the case of the highest x-gum concentration, after 2 d, the weight losses were 22% and 46% for G18/0.4 and G15/0.4, respectively. In the case of G18/0.4, the complete erosion occurred in 8 d.

#### 2.1.3. Leakage Evaluation

**Figure 4:**
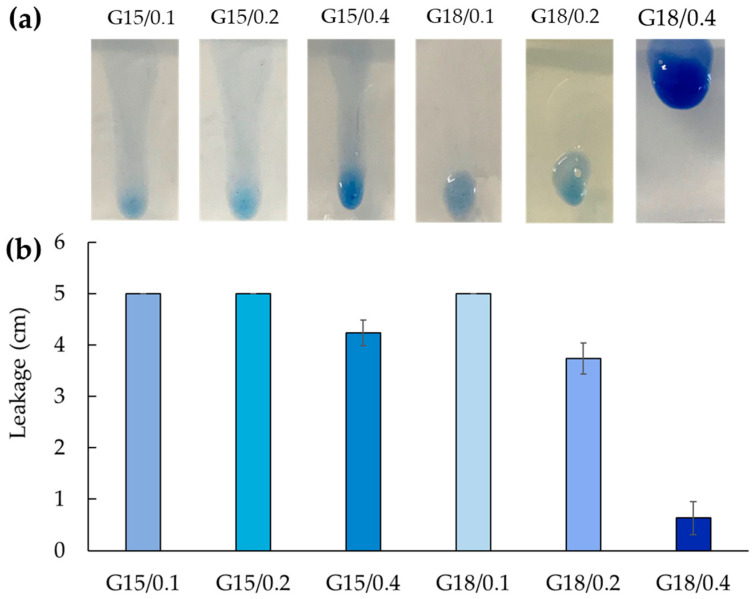
Comparative leakage test performed on formulations colored by Coomassie blue dye for imaging. Images (**a**) and leakage distances (**b**) were taken 10 s after placing the formulations on the slides. Data are the mean of three experiments ± s.d.

The main disadvantage of p-407 gel topical administration is related to its poor mucoadhesive property, resulting in short permanence on the site of application, due to a rapid dissolution in contact with body fluids, such as the vaginal [37,38,39]. In this respect, in vitro experiments were performed to compare the behavior of the gels, mimicking their vaginal application. Particularly, to investigate leakage, which should be minimal to ensure prolonged action, the running distance of the gels over a vertical plane was measured under application on SVF agar slides at 37 °C. Figure 4 shows the obtained results.

As shown in Figure 4a,b, and reported in Table 2, G15/0.1, G15/0.2, and G18/0.1 ran the longest distance, covering the entire slide length in few seconds, followed by G15/0.4, G18/0.2, and G18/0.4. In this latter case, the gel maintained its position on the slide for more than 12 h, suggesting that the highest x-gum concentration could be suitable to improve the gel permanence on the vaginal mucosa. Thus, because of the low T_sol-gel_, the slowest erosion profile, and the minor leakage, G18/0.4 was selected for Cal loading and delivery.

### 2.2. Cal-Loading in Gels

In order to obtain a gel suitable for vaginal administration of Cal in the treatment of *C. albicans* infection, the peptide (0.25 mg/mL), whose primary structure is reported in Figure 1, was solubilized in G18/0.4 before the addition of x-gum.

The resulting G18/0.4 Cal gel was transparent and homogeneous, maintaining the same pH and physical properties of the unloaded G18/0.4 gel. The analytical evaluation of Cal titer, measured by HPLC after gel disaggregation, confirmed the total peptide loading, as expected, due to the hydrophilic character of the peptide. The gel production modality, avoiding the use of high temperature, prevented the peptide chemical degradation. The Cal titer was unvaried also after 3 months from the gel preparation, suggesting the capability of the gel to maintain the drug stability.

### 2.3. In Vitro Release Tests

The influence of gel-loading on Cal release was evaluated by comparing G18/0.4 Cal (0.25 mg/mL) and a Cal solution (0.25 mg/mL) in lactate buffer, pH 4.5 (Sol Cal), using Franz cell associated with a synthetic polymeric membrane constituted of nylon. Despite animal mucosae can yield more predictable results with respect to synthetic membranes, their use involves several drawbacks due to their availability, ethical restrictions, variable results, and risks of contamination. Conversely, FDA suggests the use of synthetic membranes by Franz cell tests to compare topical forms or for quality control, being chemically inert and commercially available, and providing highly reproducible results [49,50,51].

**Figure 5:**
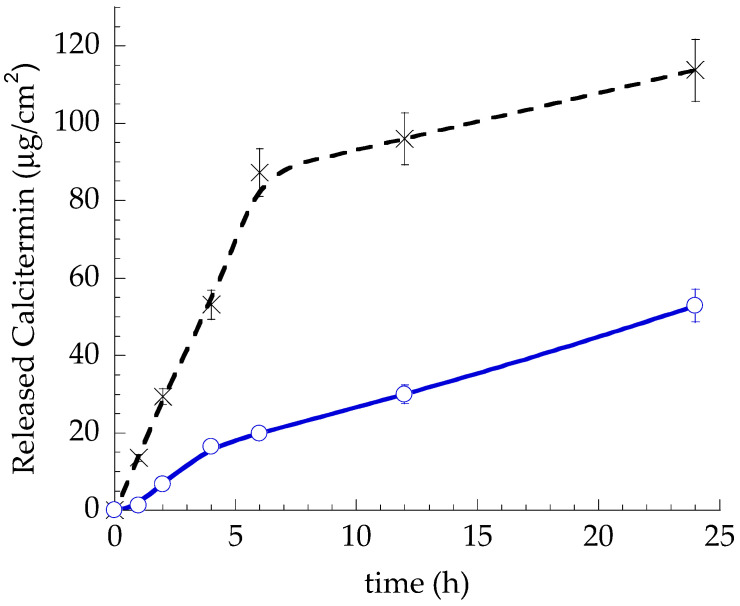
In vitro release kinetics of Cal from Sol Cal (x) and G18/0.4 Cal (o). Data corresponds to the mean values of six experiments ± s.d.

As reported in Figure 5, the peptide release from G18/0.4 Cal was more controlled with respect to Sol Cal because of the formation of a viscous gel at physiological temperature, hampering Cal release. In both cases, the peptide kinetics appeared biphasic, with a faster release within the first 6 h. After 6 h, the percentage amounts of Cal released with respect to the total amount of peptide in the formulations were 8% and 35%, in the case of G18/0.4 Cal and Sol Cal, respectively. After 24 h, the Cal released reached 21% and 45%, respectively, for G18/0.4 Cal and Sol Cal. The Cal release parameters were calculated from the slope of the linear portion of the profiles, obtained from plotting the Cal release over the square root of time (1–6 h), as reported in Figure S2 and in Table 3.

The in vitro release profile was fitted to zero order, first order, and Higuchi models to investigate the Cal release mechanism from G18/04 Cal. Data are reported in Figure S4 and Table S2. The highest R^2^ value 0.9974 suggested that the Cal release followed the Higuchi order model [52].

### 2.4. Microbiological Evaluation

To assess the anticandidal effectiveness of Cal and the effect of its loading in gels, the antifungal activity of Sol-Cal or G18/0.4-Cal was evaluated by two different tests at pH 4.5 to mimic the vaginal environment.

#### 2.4.1. Antifungal Activity of Cal in Aqueous Buffer

To assess the Cal antifungal activity, the peptide was solubilized in PBS pH 4.5 (AB Cal) at 0.125, 0.0625, and 0.0312 mg/mL, based on previously published reports [13,14].

Briefly, *C. albicans* suspensions with known titer were cultured for 3 and 24 h in the presence of the antimicrobial compound at the indicated concentrations, and the residual yeast amount was measured by CFU count on TSA plates, after further 24 h of incubation without antimicrobials. The obtained results are summarized in Table 4.

The results showed a remarkable reduction of *C. albicans* CFU number in the presence of Cal in PBS compared to controls, consisting of *C. albicans* grown in PBS pH 4.5. The reduction was dose-dependent and time-dependent. In fact, although Cal action started to be evident after 3 h of incubation, at this timepoint, a significant reduction was detected only at the highest Cal concentration (−18%, *p* < 0.01). Lower Cal concentrations were less active and did not provide significant reduction of *C. albicans* CFU number (−5% and −13% at 0.031 and 0.062 mg/mL, respectively).

**Figure 6:**
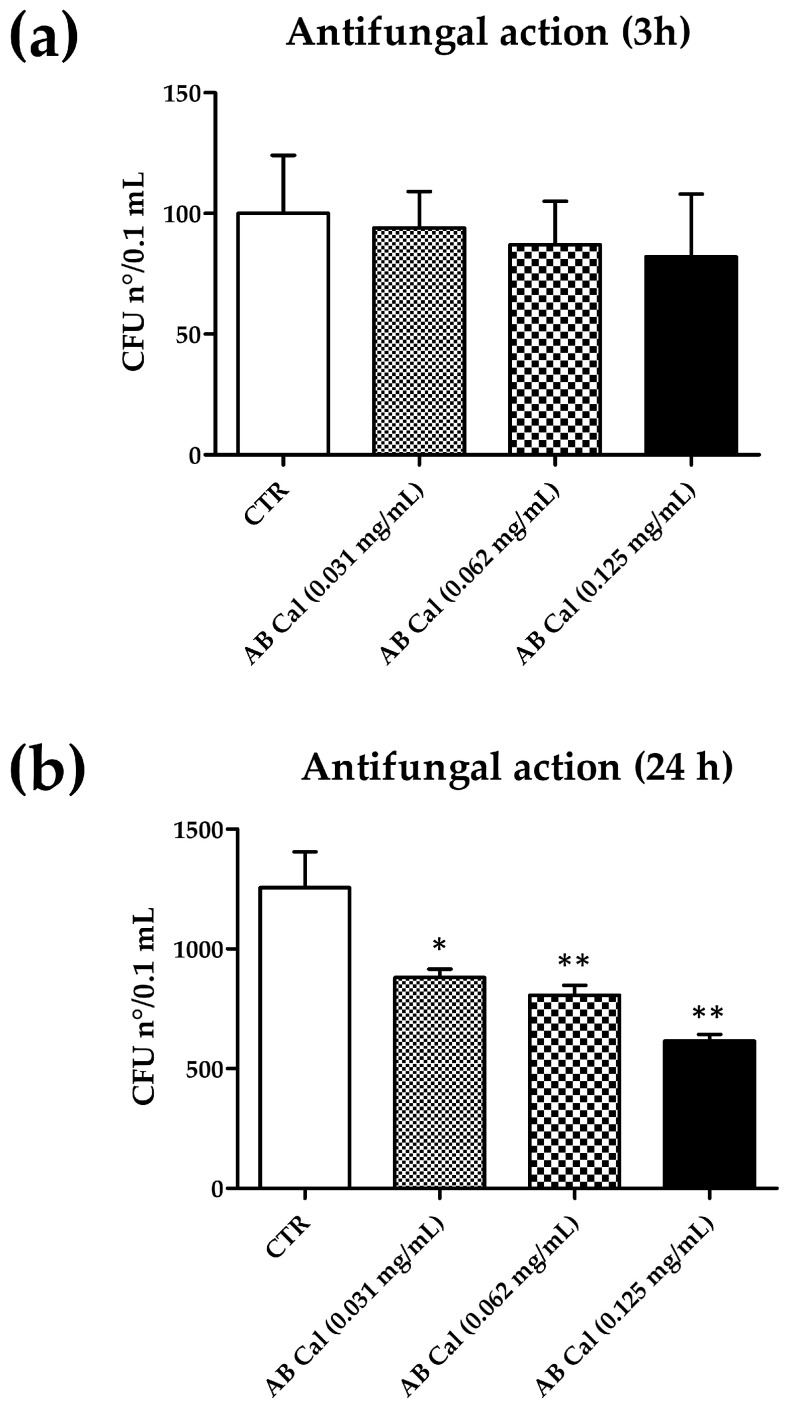
Antimicrobial activity of Cal peptide in aqueous buffer (AB). (**a**) Effect after 3 h of incubation; (**b**) Effect after 24 h of incubation. CTR, control yeast, receiving AB buffer without Cal; CFU, colony forming units. Results are expressed ad mean value of CFUs ± SD obtained in triplicate samples from two independent experiments; * *p* value ≤ 0.05; ** *p* value ≤ 0.01.

After 24 h of contact, the reduction was more evident and AB Cal reduced *C. albicans* CFUs of 30% (*p* < 0.01), 35% (*p* < 0.01), and 51% (*p* < 0.01) as compared to controls, respectively, at 0.031, 0.062, and 0.125 mg/mL. The results obtained at both timepoints are depicted in Figure 6.

#### 2.4.2. Antifungal Activity of Cal in Gel

In order to assess the influence of Cal loading in gel on its antifungal activity, the inactivation assays were performed on G18/0.4 Cal. Briefly, *C. albicans* suspensions with known titer were used to inoculate gel samples at pH 4.5 at the same concentrations used for the assays performed in the aqueous buffer. Samples were incubated for 3 and 24 h at 37 °C, and the residual yeast amount was measured by CFU count, as performed in the previous assays. The results are summarized in Table 5.

The results showed that G18/0.4 Cal induced a significant reduction of Candida CFUs at all the tested concentration and timepoints, as compared to the untreated controls consisting of yeasts cultured in the empty gel (G18/0.4). As already observed in assays performed in PBS, the inhibitory activity of Cal was dose- and time-dependent. However, when using gels, the inhibition of Candida growth by Cal was already evident after 3 h of incubation, with a CFU decrease corresponding to −29% (*p* < 0.05), −32% (*p* < 0.01), and −46% (*p* < 0.01) at 0.031, 0.064, and 0.125 mg/mL, respectively.

**Figure 7:**
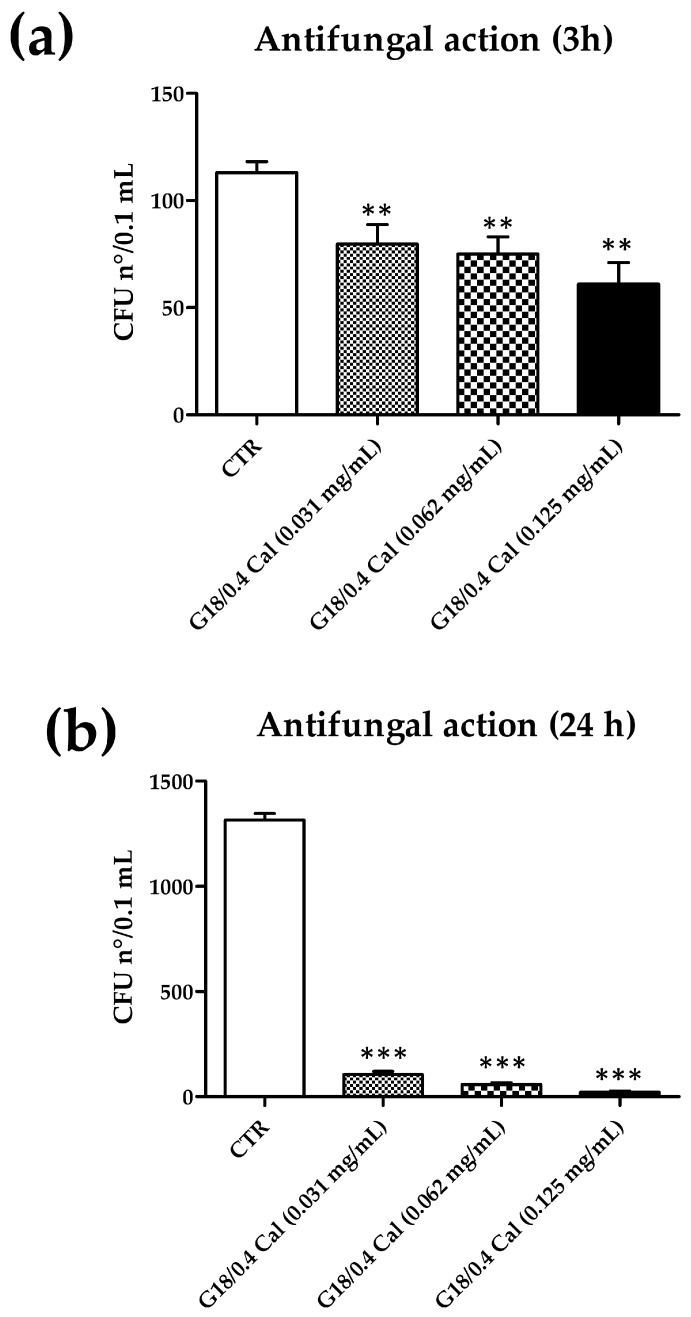
Antifungal activity of G18/0.4 Cal. (**a**) Effect after 3 h of contact. (**b**) Effect after 24 h of contact. CTR, control yeasts receiving G18/0.4 gel without Cal; CFU, colony forming units. Results are expressed as mean value of CFUs ± s.d. obtained from triplicate samples from two independent experiments; ** *p* value < 0.01; *** *p* values < 0.001.

After 24 h of incubation, the activity of Cal was further increased, showing reduction values corresponding to 92%, 96%, and 98% compared to controls (all values, *p* < 0.0001), induced by 0.031, 0.062, and 0.125 mg/mL of Cal, respectively. The results obtained at both time points are shown in Figure 7.

Cal showed good antifungal activity in all the assay types performed. The effect was dose- and time-dependent. It was detectable after 3 h of contact between the yeast *C. albicans* and Cal, but it was more evident after 24 h of contact. At both timepoints, the action increased with Cal concentration, reaching an almost total inhibition of *Candida* growth with the highest concentration at 24 h (−98% of yeast CFUs). Notably, Cal action was remarkably increased in gel compared to aqueous buffer (AB Cal), at both tested timepoints: at 3 h the highest Cal concentration reduced *Candida* CFU of 18% in PBS and 46% in gel, and at 24 h the reduction was 51% in PBS and 98% in gel. The observed differences were statistically significant and may be linked to the different physical characteristics of the medium in the two different assays, liquid for buffer samples and semisolid for gel samples, which in the last condition may allow for maintaining the antifungal peptide in close contact with yeast cells, increasing its antifungal action.

## 3. Discussion

This investigation enabled us to select a smart biocompatible gel for Cal delivery on vaginal mucosa. Particularly, the formulative study evidenced that p-407, 18% and x-gum 0.4% allowed us to obtain a gel with rheological behavior, erosion, and leakage parameters long lasting and suitable for easy applicability on the vaginal mucosa. In an aqueous solution, with increasing temperature, p-407 molecules aggregate in micelles, due to dehydration of the poly (propylene oxide) part at the core of the polymer, with an outer surface of hydrated polyethylene oxide chains [26]. Above the transition temperature, p-407 micellar solution undergoes a huge increase in viscosity due to configurational changes of the micelles in solution, resulting in an ordered packing of the micelles, and consequently, in a thermo-reversible gelation [27]. In this situation unbound water is available at the hydrophilic polyethylene oxide regions of the micelles [53]. Thereby, x-gum added to a p-407 solution at low temperature can be hydrated by water molecules. The water molecules bound to x-gum are thus subtracted from the interaction with p-407, leading to the dehydration of the polymer. In this context the presence of x-gum could indirectly promote the interaction between the p-407 micelles, finally leading to a decrease of the gel transition temperature [40,41].

In the present study, in vitro erosion and leakage tests were employed preliminarily to select the gel polymer concentrations [50]. The erosion test demonstrated the role of x-gum in controlling the disaggregation of the p-407 gel matrix in aqueous media. It is suggested that the presence of x-gum modifies the p-407 gel network, possibly increasing the entanglement and rigidity of the gel structure, thus hindering the disaggregation of the polymeric micellar network and dissolution of the polymer chains [26]. The results of the leakage test well agreed with rheological and erosion findings, confirming that the highest x-gum concentration was required to improve the physical properties of the p-407 network in water. Notably, to study the gel leakage, we preferred to employ an in vitro model based on agar slide mimicking the vaginal mucosa [50], instead of using animal samples, avoiding the variability drawbacks inevitably related to ex-vivo animal samples, that could result in a critical reproducibility. In a further study, porcine mucosa would be used to confirm the muco-adhesiveness of the Cal loaded selected gel.

The in vitro release test based on Franz cell, employed as a sensitive and discriminating method generally responsive to physicochemical changes in semisolid drug products [49], confirmed the capability of G18/0.4 Cal to control Cal release. Indeed, the Cal loading in the gel resulted in a 4-fold reduction of release rate, suggesting that the p-407 and x-gum tortuous network contributes to considerably restrain the peptide release, as compared to the simple solution of Cal. The mechanism of Cal release, followed the Higuchi’s square root model, as previously found in the case of semi-solid dosage forms [54,55]. This release model describes a system in which the dissolved drug is released from the carrier by diffusion, depending on the formulation characteristics [56]. Therefore, Cal release from G18/0.4 was mainly controlled by its diffusion from the gel matrix.

With regard to the antimicrobial activity, of note, the antifungal assays demonstrated a certain Cal capability to inhibit *C. albicans* growth in vitro at pH 4.5, halving the fungal titer in 24 h, suggesting its potential use for the treatment of *C. albicans* vaginal infections. More interestingly, a significant improvement of Cal antifungal activity was observed in the case of G18/0.4 Cal, compared to the aqueous buffer, leading to almost total inhibition of yeast growth (−98%) in 24 h of contact.

Remarkably, an increased antifungal activity by gel inclusion has been shown for several potential antifungal compounds other than Cal, confirming our present findings [57,58,59,60,61,62,63,64]. For instance metal ions [58], and conventional fungicides such as miconazole and voriconazole, showed an increased action by gel inclusion also in vivo, in animal models [62,64]. Several compounds of natural origin, such as tea tree or other oils, were reported to have a better antifungal activity when included in gel preparation [60]. In addition, gel formulations specifically addressed to treat vaginal dysbiosis (Respecta^®^ Balance Gel, RBG, marketed as an adjunct to probiotic treatment), showed a high inhibitory action against diverse *Candida* strains, including *C. albicans* and *C. glabrata,* as well as against Gram-negative and gram-positive vaginal bacteria [59]. In this perspective, the behavior of Cal observed in this pilot study suggests that its inclusion in G18/0.4, may improve the peptide antimicrobial activity.

Although Cal MIC and MFC were not determined by the methods used in the present study, the collected results confirmed previous data showing the low antifungal activity of Cal when used alone, whereas an antifungal MIC value corresponding to 1 µg/mL was measurable in the case of Cal combined with Cu^2+^ or Zn^2+^ ions [14,65]. This suggests that the addition of metal ions to Cal may substantially increase its activity, potentially providing a rapid and total yeast inhibition in gel formulations. In this respect the anticandidal action of G18/0.4 loaded with Cal/Zn complex should be investigated in future studies.

## 4. Conclusions

The increasing use of antifungal drugs in prophylaxis and treatment of invasive candidiasis has led *Candida* species to evolve a large number of resistance mechanism. In this respect, new antifungal agents, such as Cal, may hold promise as adjunctive therapies, possibly preventing the development of resistance or allowing treatment of strains resistant to conventional antifungals. However, further studies will be needed to understand the mechanism of Cal antifungal action and the efficacy of G18/0.4 Cal in animal models. As well, other *Candida* species strains should be assayed to assess a generalizable action of Cal against infectious fungi. Lastly, testing the Cal activity on drug-resistant clinical isolates of *Candida* species may provide the proof of Cal potential in the treatment of drug-resistant fungal strains.

## 5. Materials and Methods

### 5.1. Materials

Calcitermin (Cal) was purchased from KareBay Biochem (Monmouth Junction, NJ, USA) with a certified purity of 98%. The copolymer poly(ethylene glycol)-block-poly(propylene glycol)-block-poly(ethylene glycol) poloxamer 407 (p-407) (PEO98-POP67-PEO98), xanthan gum from Xanthomonas campestris (x-gum) composed of a β-(1→4)-D-glucopyranose glucan backbone with side chains of (1→3)-α-D-mannopyranose-(2→1)-β-D-glucuronic acid-(4→1)-β-D-mannopyranose on alternating residues, average molecular weight of about 2000 kDa, and Coomassie brilliant blue G 250 were purchased from Merck (Milan, Italy). Nylon membranes (2.5 cm diameter, pore size 0.2 μm) and nylon syringe filters (0.22 μm pores) were purchased from Merck (Milan, Italy). Solvents were of HPLC grade, and all other chemicals were of analytical grade.

### 5.2. Gel Preparation

P-407 hydrogel was prepared according to the “cold method” [50]. Briefly, p-407 was gradually added to a cold lactate buffer solution at pH 4.5 (5–10 °C) under magnetic stirring up to a final concentration of 15 (G15) or 20 (G20) % *w*/*w* p-407. The resulting gels were sealed and stored in a fridge at 5 °C for 12 h. Xanthan gum (x-gum) was then added to the preformed p-407 gels, under magnetic stirring at 4° C, reaching 0.1 (G15/0.1 and G18/0.1), 0.2 (G15/0.2 and G18/0.2), 0.4 (G15/0.4, and G18/0.4) or 0.5 (G15/0.5 and G18/0.5) % *w*/*w* final concentration (Table 1). The pH of the prepared gels was determined at 4 °C and adjusted to 4.5. As preliminary viscosity evaluation, a visual inspection of the formulations was performed, putting 2 mL of formulations into vials (2 cm diameter) at room temperature. The vials were then turned upside-down, noting whether the formulations were able to flow under their own weight.

### 5.3. Rheological Measurements

Rheological measurements of hydrogels were performed at 20 or 35 °C with an AR-G2 controlled-stress rotational rheometer (TA Instruments, New Castle, DE, USA) [32]. The geometry used was a sandblasted titanium plate-plate (diameter 40 mm) with a 100 µm gap. A solvent trap was used to prevent solvent evaporation during the experiments. Temperature ramps were taken from 4 °C to 50 °C with a temperature rate of 1 °C/min, controlled by a Peltier plate, with a maximum amplitude strain of 1% (in the linear regime) and a frequency of 1 Hz. A 2 min conditioning time at 4 °C was applied before starting the experiments. Measurements were performed at least three times for each sample to ensure reproducibility.

### 5.4. Erosion and Leakage Tests

Erosion of hydrogels was tested by a gravimetric method [66]. To perform the analysis, SVF, pH 4.5, was prepared dissolving NaCl (3.51 g), KOH (1.4 g), Ca(OH)_2_ (0.22 g), bovine serum albumin (0.018 g), lactic acid (2.00 g), acetic acid (1.00 g), glycerol (0.16 g), urea (0.4 g), and glucose (5.00 g) in 900 mL of distilled water. Five hundred micrograms of each hydrogel were poured into an Eppendorf tube with a known weight (W_epp_) and heated at 37 °C. Afterwards, 1 mL SVF was carefully laid over the surface of the gel and the system was maintained at 37 °C. At regular time intervals the whole supernatant was removed, the system was weighted again (Wt) and then refilled with 1 mL of fresh SVF.

The remaining gel weight for each formulation (W_gel_) was calculated according to Equation (1):

(1) $$\mathrm{Wgel}=\frac{\mathrm{Wt}-\mathrm{Wepp}}{0.5}\times 100,$$

where Wt is the weight of the Eppendorf tube + the weight of the gel at the different time intervals t, and 0.5 is the weight of the gel at the beginning of the experiment. The erosion profiles were obtained by plotting W_gel_ values against time. The experiments were repeated three times, calculating the mean values ± s.d.

To test the in vitro leakage of the formulations, hydrogels were colored by dissolving Coomassie blue (0.05% *w*/*w*) in the preformed gels. SVF agar slides were prepared adding agar (1.5% *w*/*w*) to SVF, under magnetic stirring at 95 °C, until solubilization. After cooling, the resulting gel was then cut to obtain rectangular agar slides. For leakage test, 50 mg of each colored gel were placed onto one end of the SVF agar slides. The slides were then vertically put at an angle of 90 °C and maintained at 37 ± 1 °C. The running distance of the gel along the slide was measured 10 sec after the formulation placement. Gel leakage, expressed as the distance traveled by the gel, was measured three times in independent experiments, calculating the mean values ± s.d.

### 5.5. Cal-Loaded Gels Preparation

Cal-loaded gels were obtained through the direct Cal solubilization (0.25 mg/mL) in the preformed p-407 gel, before adding x-gum. As control, for in vitro release study Sol Cal-was prepared solubilizing Cal 0.25 mg/mL in lactate buffer 20 mM, pH 4.5.

#### Evaluation of Cal Content in Gels

The content of Cal loaded in hydrogels was determined by gel dilution with water in a 1:10 *v*/*v* ratio. The obtained dispersions were maintained under magnetic stirring for 30 min and filtered by nylon syringe filters (0.2 µm pores). The content of Cal was evaluated via HPLC, as below reported.

### 5.6. In Vitro Release Test

Cal in vitro release was evaluated using Franz type glass cell assembled with a nylon membrane (GNWP, 0.2 μm pore size, Millipore, MA, USA). Briefly, dried membranes were rehydrated by immersion in lactate buffer (pH 4.5) at room temperature for 1 h before being fixed in Franz cells with an exposed surface area of 0.78 cm^2^. The receptor compartment of the cell was filled with 5 mL of lactate buffer (pH 4.5) stirred at 500 rpm by a magnetic bar, and thermostated at 37 ± 1 °C during the experiments. Five hundred microliters of Cal-loaded gel (0.25 mg/mL) or 500 μL of Sol Cal (0.25 mg/mL) were placed on the membrane surface in the donor compartment. At predetermined time (0, 1, 2, 4, 6 or 24 h) samples (0.5 mL) of receptor phase were withdrawn and analyzed by HPLC as reported below to evaluate the Cal content. Each removed sample was replaced with an equal volume of simple receptor phase.

The amount of Cal released (μg/cm^2^) was plotted against time to obtain release profiles. To calculate flux and release rate parameters, the amount Cal released was plotted against the square root of time (1–6 h), considering the flux of Cal as the slope of the cumulative amount of Cal released versus time. The release rate was obtained by dividing the flux by the Cal concentration in the formulation (0.25 mg/mL). Cal release kinetics were determined six times in independent experiments, calculating the mean values ± s.d.

The release kinetics parameters were evaluated by mathematical models (KinetDS, Aleksander Mendyk) [52], as reported in Table S2. R^2^ values were taken as the indicator of the fit level.

### 5.7. HPLC Procedures

HPLC analyses were performed using PerkinElmer, Series 200 HPLC Systems equipped with a micro-pump, an autosampler, and a UV detector operating at 200 nm. A stainless-steel C-18 reverse-phase column (15 × 0.46 cm) packed with 5 µm particles (Hypersil BDS C18 Thermo Fisher Scientific S.p.A., Milan, Italy) was eluted at a flow rate of 1 mL/min, with a mobile phase containing acetonitrile/water/trifluoroacetic acid 20:80:0.1 *v*/*v*/*v*. Cal retention time was 8 min. The method was validated for linearity, as reported in Supplementary Materials.

### 5.8. Antifungal Activity

The potential antifungal activity of Cal was investigated against *C. albicans* ATCC 10231 strain (American Type Culture Collection, ATCC, Thermo Scientific, Milan, Italy). The yeast was propagated in Tryptic Soy Broth (TSB; Biolife, Milan, Italy) or Tryptic Soy Agar (TSA, Biolife, Milan, Italy) plates incubated at 37 °C.

The antifungal activity of Cal was assessed against *C. albicans* at a final concentration corresponding to 0.125–0.062–0.031 mg/mL, based on preliminary tests and previous results obtained against different microorganisms [13,14]. Two in vitro assay types were run in parallel, to assess, respectively, the activity of Cal when diluted in phosphate buffer saline (PBS) at pH 4.5 and the activity of Cal when included in gels at pH 4.5. The acid pH value was chosen to mimic the vaginal environment. Positive and negative controls were also included in the assays, corresponding, respectively, to PBS or gel with or without the yeast strain. Prior to the assays, the validation of method regarding the growth of the used *C. albicans* strain at low pH was performed. In short, the examined strain was incubated at 37 °C for 24 h in TSB at pH 7.0 and 4.5, showing no significant influence of pH value on *Candida* growth.

Briefly, for tests carried out in aqueous buffer (PBS), the yeast *C. albicans* was cultured overnight at 37 °C under stirring in TSB and then sub-cultured by 1:10 dilution in TSB and further incubation until reaching an OD_600nm_ = 0.3–0.4, as measured by spectrophotometric reading, using a GENESYS 10S UV-Vis spectrophotometer (Thermo Scientific, Milan, Italy), which corresponded to 0.9–1.2 × 10^7^ colony forming units (CFU) per mL. The culture was then centrifuged at 4500 rpm for 10 min in a SL 8 centrifuge (TX-150 Rotor) (Thermo Scientific, Milan, Italy), and the cellular pellet was washed with PBS pH 4.5 to remove any residual culture medium. Pelletized yeasts were then suspended in 10 mL of the same buffer (PBS, pH 4.5), then the yeast suspension was diluted in PBS pH 5.4 to obtain a final concentration corresponding to 10^4^ CFU/mL. Aliquots of 0.1 mL (corresponding to 10^3^ CFU) were then seeded in each well of a 96-wells plate, and 3 µL of TSB at pH 4.5 were added to each well to obtain a final 1.5% TSB concentration in the culture medium. Cal was serially diluted in PBS pH 4.5 to obtain final concentrations corresponding to 0.257, 0.128, and 0.064 mg/mL. One hundred µL of the prepared Cal solutions were then added to each well, obtaining the final 0.128, 0.064, and 0.032 mg/mL Cal concentrations in a 0.2 mL volume. PBS alone was also included as a control. The plates were incubated a 37 °C on a platform rocker with slow stirring for 3 and 24 h. At the end of the incubation times, an aliquot portion of 0.1 mL was taken from each sample and diluted in PBS pH 4.5 at, respectively, 1:10 at 3 h and 1:200 at 24 h. Finally, 0.1 mL of such diluted suspensions were seeded in Tryptic Soy Agar (TSA) plates and incubated at 37 °C for 24 h for CFU count. Samples were assayed in triplicate in two independent experiments, then 0.1 mL were taken, properly diluted as described for the previous assay, and seeded on TSA plates. Plates were incubated for further 24 h at 37 °C, afterwards *C. albicans* CFUs were enumerated.

In the second assay type, the yeast *C. albicans* culture was obtained as described for the previous assay, diluted to obtain a final concentration corresponding to 2 × 10^4^ CFU/mL. Aliquots of 0.5 mL (corresponding to 10^4^ CFU) were seeded in every well of a 24-wells plate, then immediately covered with 0.5 mL of gel containing 0.250, 0.125, or 0.062 mg/mL of Cal, obtaining final Cal concentrations of 0.125, 0.0625, and 0.0312 mg/mL in 1 mL of final volume. The plates were then incubated a 37 °C on a platform rocker with gentle stirring for 3 and 24 h, then aliquots of 0.1 mL were taken from each sample, properly diluted in PBS pH 4.5, as described for the previous assay, and seeded in TSA. Plates were then incubated at 37 °C for 24 h for CFU count. Samples were assayed in triplicate in two independent experiments in both assay types. MIC and MFC were not determined, since the values obtained in previously published studies were taken as a reference [14].

### 5.9. Statistical Analyses

Student’s *t*-test was used for statistical analyses, and a *p* value ≤ 0.05 was considered significant.

## Figures and Tables

**Table 1:** Gel compositions and pH.

Gels	Poloxamer 407	Xanthan Gum	Lactate Buffer *	pH
G15/0.1	15	0.10	84.85	4.4
G15/0.2	15	0.20	84.80	4.5
G15/0.4	15	0.40	84.60	4.5
G18/0.1	18	0.10	81.90	4.4
G18/0.2	18	0.20	81.70	4.4
G18/0.4	18	0.40	81.60	4.6

* Lactate buffer 20 mM.

**Table 2:** Rheological parameters and leakage of the indicated gels.

Gels	T_sol-gel_ ^§^ (°C)	Determination of T_sol-gel_	Leakage (cm) *
G15/0.1	22.7 ± 2.3	G′-G″ crossover	5.00 ± 0.00
G15/0.2	23.7 ± 5.1	onset of sharp G′ increase	5.00 ± 0.00
G15/0.4	25.4 ± 1.0	onset of sharp G′ increase	4.23 ± 0.25
G18/0.1	21.4 ± 2.7	G′-G″ crossover	5.00 ± 0.00
G18/0.2	20.5 ± 2.4	onset of sharp G′ increase	3.73 ± 0.30
G18/0.4	18.6 ± 4.9	onset of sharp G′ increase	0.63 ± 0.32

^§^ transition temperature; * gel running distance along the slide. Data are the mean of 3 experiments ± s.d.

**Table 3:** Release parameters of Cal loaded in the indicated forms.

Formulation	Flux ± s.d. (μg/cm^2^/h)	Cal (mg/mL)	Release Rate (cm/h) × 10^−3^	Reduction Ratio ^1^
Sol-Cal	13.31 ± 1.50	0.25	53.24 ± 6.00	-
G18/0.4-Cal	49.51 ± 0.71	0.25	198.04 ± 2.84	3.72

^1^: reduction of Cal release rate with respect to Sol-Cal; data are the mean of 6 independent Franz cell experiments.

**Table 4:** Antifungal activity of Cal in aqueous buffer (AB).

	Incubation Time	
	3 h	24 h
Condition	CFU n°/sample (*)	CFU n°/sample (*)
AB (CTR)	100 ± 24	1.255 ± 150
AB Cal (0.031 mg/mL)	94 ± 15 (−5%)	880 ± 35 (−30%)
AB Cal (0.062 mg/mL)	87 ± 18 (−13%)	807 ± 41 (−35%)
AB Cal (0.125 mg/mL)	82 ± 26 (−18%)	616 ± 27 (−51%)

(*) The results are expressed as mean ± s.d. of enumerated CFUs CFUs ± s.d. obtained in triplicate samples from two independent experiments; each sample corresponded to 0.1 mL of yeast suspension diluted in PBS 1:10 and 1:20 at 3 and 24 h, respectively; percentages of inhibition are also reported in parentheses. CTR, control sample, corresponding to AB without Cal.

**Table 5:** Antifungal activity of the indicated gels.

	Incubation Time	
	3 h	24 h
Condition	CFU n°/sample (*)	CFU n°/sample (*)
G18/0.4 (CTR)	113.0 ± 5.2	1.316 ± 31.0
G18/0.4 Cal (0.031 mg/mL)	79.7 ± 9.1 (−29%)	105.1 ± 15.0 (−92%)
G18/0.4 Cal (0.062 mg/mL)	75.0 ± 8.0 (−32%)	57.7 ± 9.0 (−96%)
G18/0.4 Cal (0.125 mg/mL)	61.0 ± 10.0 (−46%)	22.0 ± 4.5 (−98%)

(*) The results are expressed as mean ± S.D. of enumerated CFUs CFUs ± s.d. obtained in triplicate samples from two independent experiments; each sample corresponded to 0.1 mL of yeast suspension diluted in PBS 1:10 and 1:20 at 3 and 24 h, respectively; percentages of inhibition are also reported in parentheses. CTR, control sample, corresponding to pure G18/0.4 gel without Cal.

## Data Availability

All the data supporting reported results are included in the article and in its Supplementary Materials.

## Supplementary Materials

The following supporting information can be downloaded at: https://www.mdpi.com/article/10.3390/gels9020165/s1, Figure S1: G18/0.4 before (a) and after (b) vial inversion at room temperature; Figure S2: Cal calibration profile; Figure S3: In vitro release kinetics of Cal from Sol Cal (x) and G18/0.4 Cal (o). Data corresponds to the mean values of six experiments ± s.d.; Figure S4: Fitting of Cal release to zero-order (a), first-order (b), and Higuchi (c) kinetic models for G18/0.4-Cal; Table S1: Predefined acceptance criteria and results for the IVRT method validation; Table S2: Kinetic release data of Cal.
