# Supplementary material for: Calcitermin-Loaded Smart Gels Activity against Candida albicans: A Preliminary In Vitro Study
Source: Gels. 2023 Feb 18;9(2):165. doi: 10.3390/gels9020165 (PMC9957098; doi:10.3390/gels9020165)
Supplement: Supplementary file 1 [file gels-09-00165-s001.zip › gels-2212956-supplementary.pdf]

Supplementary data

Calcitermin-Loaded Smart Gels Activity against *Candida albicans*: A Preliminary In Vitro Study

Denise Bellotti [1,2,†], Maria D'Accolti [3,†], Walter Pula [3], Nicolas Huang [4], Fanny Simeliere [4], Elisabetta Caselli [3,*], Elisabetta Esposito [3,*] and Maurizio Remelli [3]

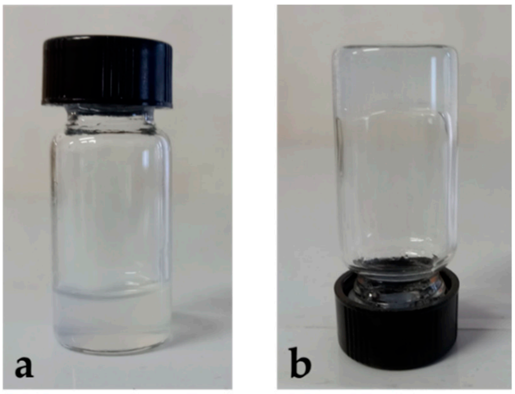

Figure S1. G18/0.4 before **(a)** and after **(b)** vial inversion at room temperature.

S.1. In Vitro Release Test (IVRT)

Each IVRT run was performed with six Franz cells operating in parallel. The receptor medium was stirred at 500 rpm, and the temperature was maintained at 37 ± 1 °C. The Franz cell system was allowed to equilibrate for approximately 30 min. Samples of the receptor medium, consisting of lactate buffer, were collected from each of the 6 cells simultaneously at 0, 1, 2, 4, 6, 12 and 24 h after application of Sol Cal or 0, 1, 2, 4, 6, 12 and 24 h after application of G18/0.4.

S.1.1. HPLC-UV method validation

The Cal samples were analyzed using PerkinElmer, Series 200 HPLC Systems equipped with a micro-pump, an autosampler, and a UV detector operating at 200 nm. A stainless-steel C-18 reverse-phase column (15 × 0.46 cm) packed with 5 μm particles eluted at a flow rate of 0.5 mL/min, with a mobile phase containing acetonitrile/water/trifluoroacetic acid 20:80:0,1 v/v/v. The HPLC-UV method was validated according to linearity. Particularly, a set of 5 Cal calibration standards with concentrations of 0.5 (C5), 1 (C4), 5 (C3), 10 (C2) and 20 (C1) μg/mL were prepared in lactate buffer solution (receptor medium) and measured in 3 batch runs. For each of the 3 batch runs, a linear regression curve was established.

The pre-determined specification for the validation establishes that the measured concentration C_{meas} should lie within ± 15% of the nominal concentration (C_{nom}) for 75% of the standards, and at least for one standard per concentration. Additionally, the R^2 values of each regression curve calculated for each batch run should be greater than 0.95.

Table S1. Predefined acceptance criteria and results for the IVRT method validation.

Parameters	Acceptance Criteria	Results	Pass
Linearity	75% of the standards meet the following criteria: $C_{meas} \in [C_{nom} \pm 15\%]$	All standards met the acceptance criteria	yes
	$R^2 \geq 0.95$	$R^2 = 0.99$	yes

As reported in Table S1 and shown in Figure S2, the standards of Cal met the acceptance criteria for linearity.

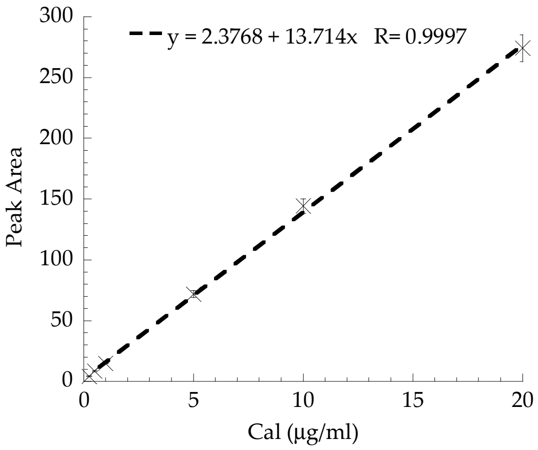

Figure S2. Cal calibration profile.

Figure S3 reports the plot of the cumulative amount of Cal released from Sol Cal and G18/0.4 Cal against the square root of time (1-6 h). The slope of the line corresponds to the flux of Cal, from which the release rate was obtained dividing the flux by the Cal concentration in the formulation (0.25 mg/mL). Cal release kinetics were determined 6 times in independent experiments, calculating the mean values ± s.d. As shown in Figure S2, the regression coefficients, were > 0.98, both for Sol Cal and G18/0.4 Cal.

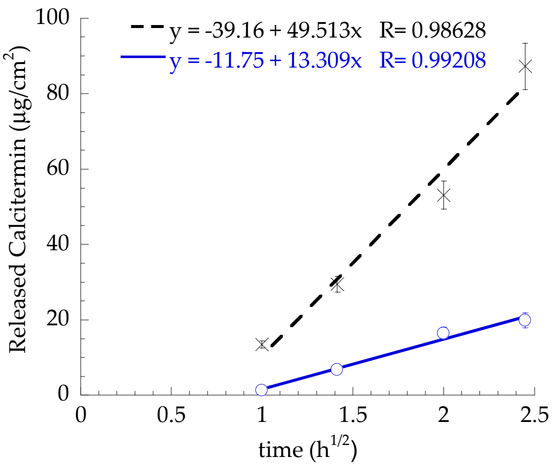

Figure S3. In vitro release kinetics of Cal from Sol Cal (x) and G18/0.4 Cal (o). Data corresponds to the mean values of six experiments ± s.d.

S.2. Kinetic Study of the Cal release

To investigate the mechanisms of Cal release, the following kinetic models were applied:

(1) The zero-order model, used for pharmaceutical dosage forms that do not disintegrate, characterized by a very slow drug release, expressed by the equation:

$$Qt = Q_0 + k0t \qquad \text{(S1)}$$

where Qt is the amount of dissolved drug at time t, Q_0 is the initial amount of drug in the solution usually (Q_0 = 0) and k_0 is the constant of zero-order release.

(2) The first-order model, used to describe the absorption and release of water soluble drugs from porous matrices, according to the equation:

$$Log_{Qt} = Log_{Q0} - k_1\, 2.303\ t \qquad \text{(S2)}$$

where Qt is the amount of dissolved drug at time t, Q_0 is the initial amount of drug in the solution, and k_1 is the constant of first-order release.

(3) The Higuchi model, used to describe the release of soluble and sparingly soluble drugs in aqueous media, from various semi-solid and/or solid matrices, expressed by the equation:

$$Qt = kHt\ 1/2 \qquad (S3)$$

where kH is the Higuchi dissolution constant, and Qt and t are the parameters described previously.

The drug release kinetics was determined by fitting the experimental data to the kinetic models. Figure S4 reports the fitting to the different kinetic models of Cal release data from G18/04 and Table S2 the kinetic data.

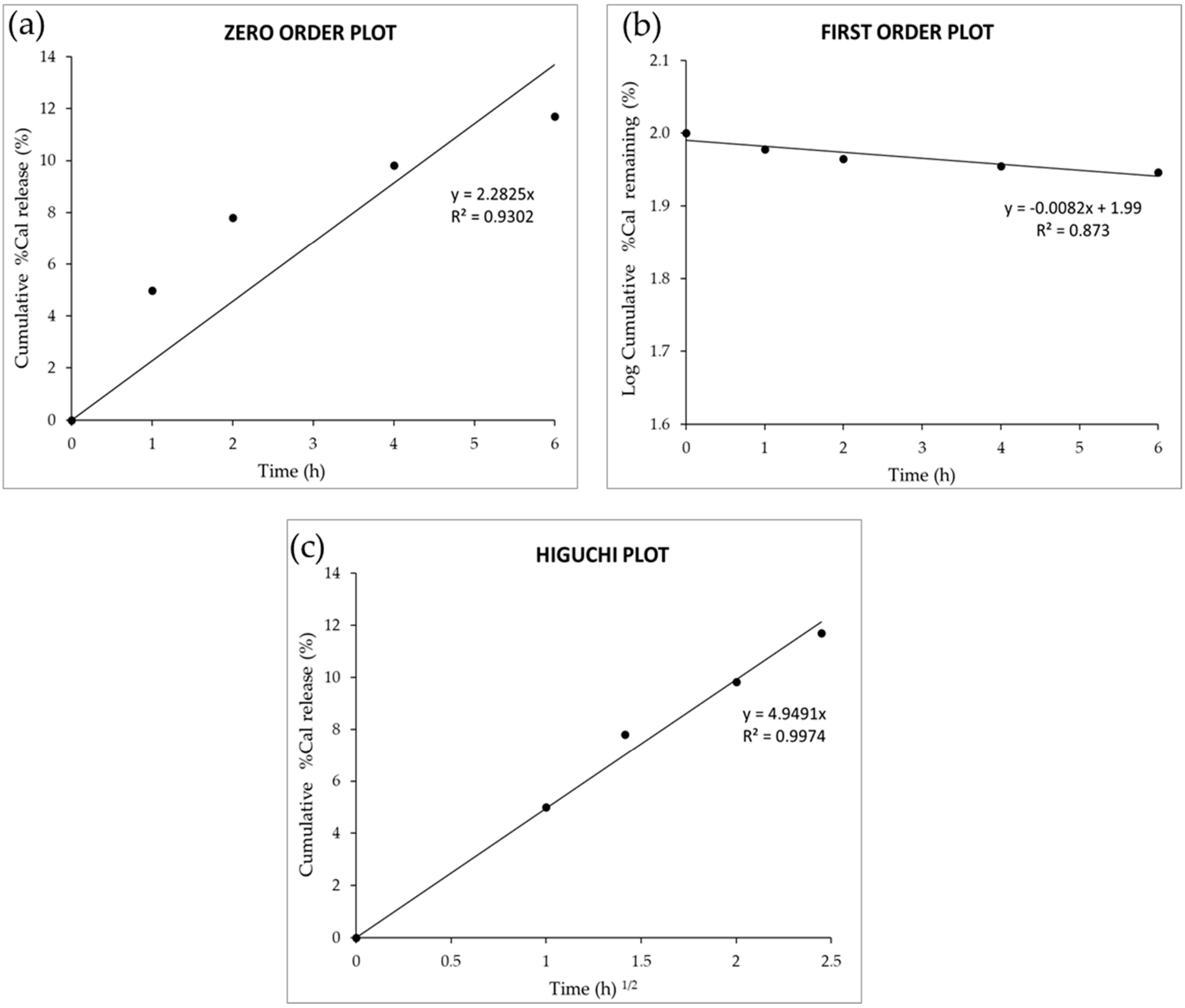

Figure S4. Fitting of Cal release to zero-order (a), first-order (b), and Higuchi (c) kinetic models for G18/0.4-Cal.

Table S2. Kinetic release data of Cal.

Formulation	Zero Order Plot (R^2)	First Order Plot (R^2)	Higuchi Plot (R^2)
G18/0.4-Cal	0.9302	0.873	0.9974
